# Extracts from Lectures on Operative Dental Surgery

**Published:** 1888-02

**Authors:** William St. George Elliott


ARTICLE VII.

### EXTRACTS FROM LECTURES ON OPERATIVE DENTAL SURGERY.

BY WILLIAM ST. GEORGE ELLIOTT, M. D., D. D. S.

I would like to say something about the principles governing the proper formation of cavities. These are general and special; the former apply to all cavities; the latter to special classes. The general principles are: First the removal of all soft decay except over the nerve in deep-seated caries. This we do mainly for physical reasons, as we cannot expect even an amalgam filling to withstand the force of mastication if it is built on a soft foundation.

When force is applied the foundation yields, and the stopping leaks as a consequence. Formerly we were taught in the text books that if we wish to preserve a tooth by stopping we must remove every trace of decay, even if in so doing we expose the nerve, otherwise the decay would perpetuate itself under the filling, Now we know that the function of a plug is the same as that of a cork in a bottle; if the cork is tight and impervious, and the neck where the cork is inserted is hard and smooth, the contents are preserved.

The second general principle is the shaping of the cavity so as to make it accessible as the stopping to be put in requires, in addition to which the interior should be so excavated as best to keep the filling in place; for example: for cohesive gold, all parts of the cavity should be accessible to the direct force applied to the plugger. Some operators take the groumd that if you cannot see all parts of the cavity by direct light, it should not be filled with gold—that I believe to be incorrect teaching; but, as with all extremes; truth is found between them. Make the cavity as accessible as the occasion calls for; better to use a little hand-pressure than unnecessarily cut away the sound tooth structure; better to work in almost inaccessible places than tire out your patient by prolonged grinding; at the same time remember that fair access is essential to good work, and shortens materially the time required for filling. I have noticed that the best operators cut the teeth the most; while the reverse is also true—the poor operator is generally saving of excavation.

The cavity should be made so as to retain the stopping, except when oxyphosphate is used. All other than the white cements have little or no cohesion to the tooth itself, and merely play the part of corks, consequently you must rely upon retaining points, undercuts, or dovetails.

The third general rule is to leave the walls of the cavity as strong as possible, to remove all thin edges of enamel, grind down all projecting points, and thoroughly smooth all epges, preferably with a fine corundum point.

Fourth general rule: See when you come to close the orifice of the cavity, when cohesive gold is used, that the gold is thoroughly beaten against the edges, and to do this effectually the edges should always be at or near a right angle to the *axis of your plugger.* With amalgam this would leave the edges thin and liable to break, consequently for that material the edges of the cavity should be made at right angles to or perpendicular to the surface of the tooth.

The special principles governing the formation of cavities will be developed as we proceed.

Let us take up first, cavities between the incisors. The first special rule is to examine carefully the labial and palatine walls of the cavity; if both are strong, then enter the cavity from the front, excavate and fill. This should always be done except when there are some epecial reasons, objection to the gold showing, &c., when some other course must be taken. It is remarkable how fair access to a cavity can be had through a very small opening, and unless the cavity is large the gold will hardly be perceptible. Opening the cavity in this way enables one thoroughly to remove all decay, to fill well and quickly; whereas if one has to do the work from behind, through the palatine opening, it is not only much more difficult, but is likely not to be thoroughly done. However, should the palatine wall be too thin to be kept, then open at that point, and show your skill by filling from the rear. Should both walls be poor and thin, better to remove them both than endanger your work by their subsequent breaking down. Then drill out a good pit in the cervical edge of the cavity, as you must get your principal support from this part, and get what undercut you can under the cutting edge. Some times a very good pit can be made between the plates of the enamel near the cutting edge. It is sometimes wise, as additional security, to drill a hole in the palatine aspect o the tooth, and, having cut under, connect it with the main cavity by a dove-tailed channel. Contouring the front teeth, unless of a very partial character, I do not advise. The gold is too conspicuous. Do not let your ambition run away with your judgment. Contour work is very easy at this part, and from a technical point of view we all admire it, but our art should be conspicuous in hiding itself. If a tooth is badly broken, I prefer rather to cut it off and pivot or crown it than to use gold. If not very badly broken away, and yet very conspicuous, I would advise white cement or gutta-percha.

Anterior cavities in the canines should be filled as incisors are; but if distal they will generally have to be opened from behind, using a pit as a starting point on either side.

Between the bicuspids decay is exceedingly common. If they are mesial, they present no difficulties, as the excavation and subsequent stopping are readily done. It is advisable, however, unless the cavity is very small, to bevel or flare the opening, so that the gold will be carried well over the walls on either side and thus in a measure prevent the recurrence of decay. The crown portion should also project forward as much as the space will permit. This is called knuckling. So that the only parts in contact with the anterior tooth are at or near the cutting edge, thus preventing the crowding of food down between the teeth, a result that is sure to follow if a V-shaped space is left. You may, however, be unable to form this contour as you would wish, by reason of limited undercut; in a close articulation, stoppings that are quite well enough anchored for ordinary positions, are often bitten quite out by the great force of mastication.

When distal cavities of bicuspids occupy our attention, our principle of proceeding is quite altered. Owing to the indirect approach to the cavity, it is necessary for us to apply our force in a less direct manner. For this reason we generally use in these cases a matrix, either a thin piece of steel placed between the teeth and wedged into position; or a band matrix with a screw to draw the band into close contact with the teeth. The Ladmore-Brunton, already mentioned, answers the purpose admirably. The use of the matrix reduces the compound to a simple cavity, for remember we have long ago abandoned the filling of a V-shaped space between the teeth to make accessible a distal cavity, instead of which we now almost invariably drill into these approximal cavities from the crown. In doing this we make a simple cavity compound, but we gain far more accessibility than we lose in other ways. Thoroughly excavating under these circumstances is easy and certain, whereas by the old way it was difficult and uncertain.

Before placing the matrix in position, the cavity should be shaped so as to get: as far as possible, direct pressure against the edges. It is practically impossible to condense gold by any other than direct pressure. You cannot do so when the walls of the cavity are parallel with the instrument, as force applied in this way rather tends to draw the gold away from than force it against the walls. To some extent we get over the difficulty by using curved or corkscrew pluggers, directing the force, as applied by the mallet, by the hand holding the plugger. Still it is always preferable to use the direct force itself rather than change the direction by the fingers.

Where it is practicable, so shape the distal cavities in bicuspids that they will be smaller across at the cervical wall than at the crown, trumpet shaped in fact. In this way you can get access and consequent solidity, but of course you have no anchorage. This you must get inside the vertical walls either by grooves, pits, or channels.

In regard to the cavities in molars, they are so various that it is hard to lay down any principle. Approximal cavities are treated as if they were bicuspids, while crowu fillings present no points of difficulty.

I need not remind you that the preparation of the cavity is governed by the material subsequently to be used in filling it. For cohesive gold you merely require the anchorages necessary to hold it in position, for you can build out in any direction. For tin and gold, or soft gold, you require nearly parallel walls, malleting from the distal wall forwaad. For amalgam try and avoid thin edges by removing the excess of the material, and not allowing it to go beyond the parallel walls, but for the white cements or gutta-percha it is not necessary to do even this. There is a class of cavities that we have not mentioned, and that calls for considerable skill in the use of cohesive gold. I allude to erosions. The disease is very common, and gold seems to meet the requirements better than any other material. My plan of procedure is as follows: excavate all soft dentine, and undercut with a drill and wheel bur all or nearly all around; do not rely upon pits. Then, having placed the rubber dam in position (thick dam with a very small hole), I keep the rubber above the gums by a sharp two-pronged instrument, then fill with crystal gold. The essential is dryness, and this is readily secured if you make but one hole in the rubber and with the silk force up folds between the teeth, so that there is an excess near the gum.—*The Dental Record.*

